# Direct, Specific and Rapid Detection of Staphylococcal Proteins and Exotoxins Using a Multiplex Antibody Microarray

**DOI:** 10.1371/journal.pone.0143246

**Published:** 2015-12-01

**Authors:** Bettina Stieber, Stefan Monecke, Elke Müller, Joseph Büchler, Ralf Ehricht

**Affiliations:** 1 Alere Technologies GmbH, Jena, Germany; 2 Institute for Medical Microbiology and Hygiene, Technische Universität Dresden, Dresden, Germany; 3 Infectognostics Forschungscampus Jena, Jena, Germany; 4 Alere San Diego, Inc., San Diego, California, United States of America; Naval Research Laboratory, UNITED STATES

## Abstract

**Background:**

*S*. *aureus* is a pathogen in humans and animals that harbors a wide variety of virulence factors and resistance genes. This bacterium can cause a wide range of mild to life-threatening diseases. In the latter case, fast diagnostic procedures are important. In routine diagnostic laboratories, several genotypic and phenotypic methods are available to identify *S*. *aureus* strains and determine their resistances. However, there is a demand for multiplex routine diagnostic tests to directly detect staphylococcal toxins and proteins.

**Methods:**

In this study, an antibody microarray based assay was established and validated for the rapid detection of staphylococcal markers and exotoxins. The following targets were included: staphylococcal protein A, penicillin binding protein 2a, alpha- and beta-hemolysins, Panton Valentine leukocidin, toxic shock syndrome toxin, enterotoxins A and B as well as staphylokinase. All were detected simultaneously within a single experiment, starting from a clonal culture on standard media. The detection of bound proteins was performed using a new fluorescence reading device for microarrays.

**Results:**

110 reference strains and clinical isolates were analyzed using this assay, with a DNA microarray for genotypic characterization performed in parallel. The results showed a general high concordance of genotypic and phenotypic data. However, genotypic analysis found the *hla* gene present in all *S*. *aureus* isolates but its expression under given conditions depended on the clonal complex affiliation of the actual isolate.

**Conclusions:**

The multiplex antibody assay described herein allowed a rapid and reliable detection of clinically relevant staphylococcal toxins as well as resistance- and species-specific markers.

## Introduction

Routine laboratories focus on culturing and identifying bacterial species, as well as obtaining their susceptibility profiles. Some susceptibility test results, such as oxacillin/methicillin resistance in staphylococci, vancomycin resistance in enterococci or carbapenem resistance in enterobacteria, require additional assays for confirmation due to their high relevance for therapy of individual patients, and for infection control. This can be done by molecular methods or using antibody-based assays. Molecular methods require sophisticated and expensive equipment. Currently, antibody-based tests are widely used. Examples include agglutination assays or lateral flow (LF) tests, e.g., for confirmation of the presence of modified penicillin binding protein (PBP2a) conferring oxacillin/methicillin resistance in *Staphylococcus aureus*/MRSA [1].

*Staphylococcus aureus* (*S*. *aureus*) is a common opportunistic pathogen. It colonizes approximately 30% of a healthy human population [2], but can also cause nosocomial or community-acquired infections. Clinically, *S*. *aureus* is associated with skin and soft tissue infections, food intoxications, and life-threatening diseases like pneumonia, endocarditis or septicemia. Carriers of *S*. *aureus*, in particular hospitalized, dialysis and catheter patients, show an increased risk of invasive infections [3] but a lower risk of septicemia-related death [4].

A major problem with *S*. *aureus* is the high rate of resistance to methicillin and other beta-lactam antibiotics (MRSA), especially in nosocomial settings. Normally, methicillin inhibits the cell wall synthesis of the bacteria by binding to their penicillin-binding proteins (PBPs). The gene *mecA* encodes for a modified penicillin-binding protein (PBP2a). PBP2a performs the function of PBP by synthesizing peptidoglycan, therefore methicillin cannot bind anymore [5]. In life-threatening situations it is important to rapidly detect the presence of *mecA* in order to ensure efficient, *i*.*e*., non-beta-lactam-based therapy. Additionally, MRSA-positive patients should be isolated in separate rooms to avoid a transmission to other patients. In routine diagnostics, the beta-lactam resistance, caused by PBP2a, is detected by agar diffusion or micro dilution tests, and the presence of *mecA*/PBP2a is then confirmed by either PCR, agglutination or lateral flow assays [6].

In addition to *mecA*, a highly divergent homologue, *mecC*, was recently identified [7–8]. This gene also encodes for beta-lactam resistance. Due to its low homology to *mecA*, *mecC* caused concern in diagnostics. While selective media and susceptibility tests can indicate methicillin resistance in *mecC* strains, confirmatory tests frequently do not identify them [9]. Beside *mecA/C*, some staphylococci (e.g., *S*. *sciuri* and *S*. *vitulinus*) can harbor other *mecA* alleles (“*mecA1*”) that do not encode resistance to beta lactam compounds [10–11].

MRSA has become a global problem—first in hospitals, but for approximately the last 25 years, also in non-hospitalized individuals. The so-called community-acquired MRSA (CA-MRSA) strains are often found to be more virulent than hospital-acquired MRSA (HA-MRSA), and can even infect young and otherwise healthy people. Typical properties of these clones are the presence of the smaller sized type IV or V staphylococcal cassette chromosomes *mec* (SCC*mec*) and, in many but not all strains, of the Panton Valentine leukocidin (PVL) [12],[13–24]. PVL is a leukocidin of special medical relevance that has been previously reviewed in detail [12], [13–22],[23].

Relevant virulence factors in *S*. *aureus* range from hemolysins, e.g., alpha- and beta-hemolysins (HLA, HLB) [25–29],[30], and other enzymes that digest host tissues to yield nutrients to proteins that disrupt or manipulate the host immune system [31–33]. These proteins include Superantigens, such as toxic shock syndrome toxin (TSST) [4], [34–37], staphylococcal enterotoxins (SEs) [38–41], and leukocidins. Superantigens lead to an antigen-unspecific T-cell activation followed by an immense cytokine release [42]. Currently, the detection of staphylococcal toxins relies largely on molecular methods, *i*.*e*., PCR or array hybridization [43]. These approaches are mainly restricted to research and/or reference laboratories. In routine laboratories, options for detecting staphylococcal toxins are limited, since there are no routine diagnostic tests to confirm the presence of multiple staphylococcal toxins. Enzyme-linked Immunosorbent Assays (ELISAs) or Lateral Flow tests are mainly focusing on one target only [44–45].

A multiplex test for Staphylococcal toxins could be helpful, because infections with *S*. *aureus* producing toxins should be treated differently than infections with *S*. *aureus* lacking those toxins. The presence of PVL mandates special infection control and eradication measures (HPA guideline: https://www.gov.uk/government/collections/panton-valentine-leukocidin-pvl-guidance-data-and-analysis), or a clinical condition related to PVL or TSST1 might be treated with gamma globulin and/or compounds inhibiting toxin biosynthesis (such as rifampicin, clindamycin) in addition to the standard regimen [46]. Given the clinical relevance of antibiotic resistance in *S*. *aureus* and of its various exotoxins, an assay for the detection of the respective proteins could be of high interest.

The aim of this study was to develop a new, rapid and economic fluorescence-based assay for qualitative or semi-quantitative analysis of expressed proteins, starting with clonal cultures obtained by routine laboratory procedures. A designated new reader and software were developed for the analysis of fluorescence microarray images. An antibody microarray was designed to allow simultaneous detection of PBP2a, important secreted virulence factors (TSST, PVL, SEA, SEB, HLA, HLB, Staphylokinase (SAK)) as well as a species marker that serves as a positive control (staphylococcal protein A, SPA).

In parallel to the phenotypic detection using the protein microarrays, the presence of genes and alleles coding for virulence factors was investigated during this study using DNA microarrays. A direct comparison of genotypic and phenotypic data for the targets was therefore possible.

## Materials & Methods

### Strains

In this study, 110 bacterial strains/isolates were tested. These included 105 *S*. *aureus*, 2 *S*. *epidermidis* (ATCC35984 and ATCC12228), 1 *S*. *sciuri*, 1 *S*. *capitis* and 1 *E*. *coli* (BL21-DE3, National Laboratory New York, USA) as negative control. Most of the strains/isolates originated from clinical diagnostic samples, but some well characterized reference strains were also tested. All of them were genotyped by DNA microarray hybridization (StaphyType Kit, Alere Technologies GmbH, Jena, Germany). This provided information regarding the presence or absence of relevant virulence and resistance genes, including those that encode PVL, TSST, HLA, HLB, SEA, SEB, SAK, PBP2a and Protein A, as well as the affiliation to clonal complexes and strains.

### Culture conditions

In previous experiments, different growth media were tested for strain culturing and detection of proteins [21],[25]. Based on these results, strains and isolates were incubated on Columbia Blood agar (Oxoid, Wesel, Germany) at 37°C for 18–24 h. One loop of bacterial material was inoculated into 130 μl phosphate buffered saline (1x PBS) or into 65 μl sodium hydroxide (NaOH; 100mM), respectively, and vortexed. To the NaOH suspension, 65 μl of buffered phosphate buffer (pH 5.5; 1M di-sodium hydrogen phosphate and sodium di-hydrogen phosphate) was added for neutralization (pH 7), followed by additional vortexing.

### Antibodies

**Fig 1:**
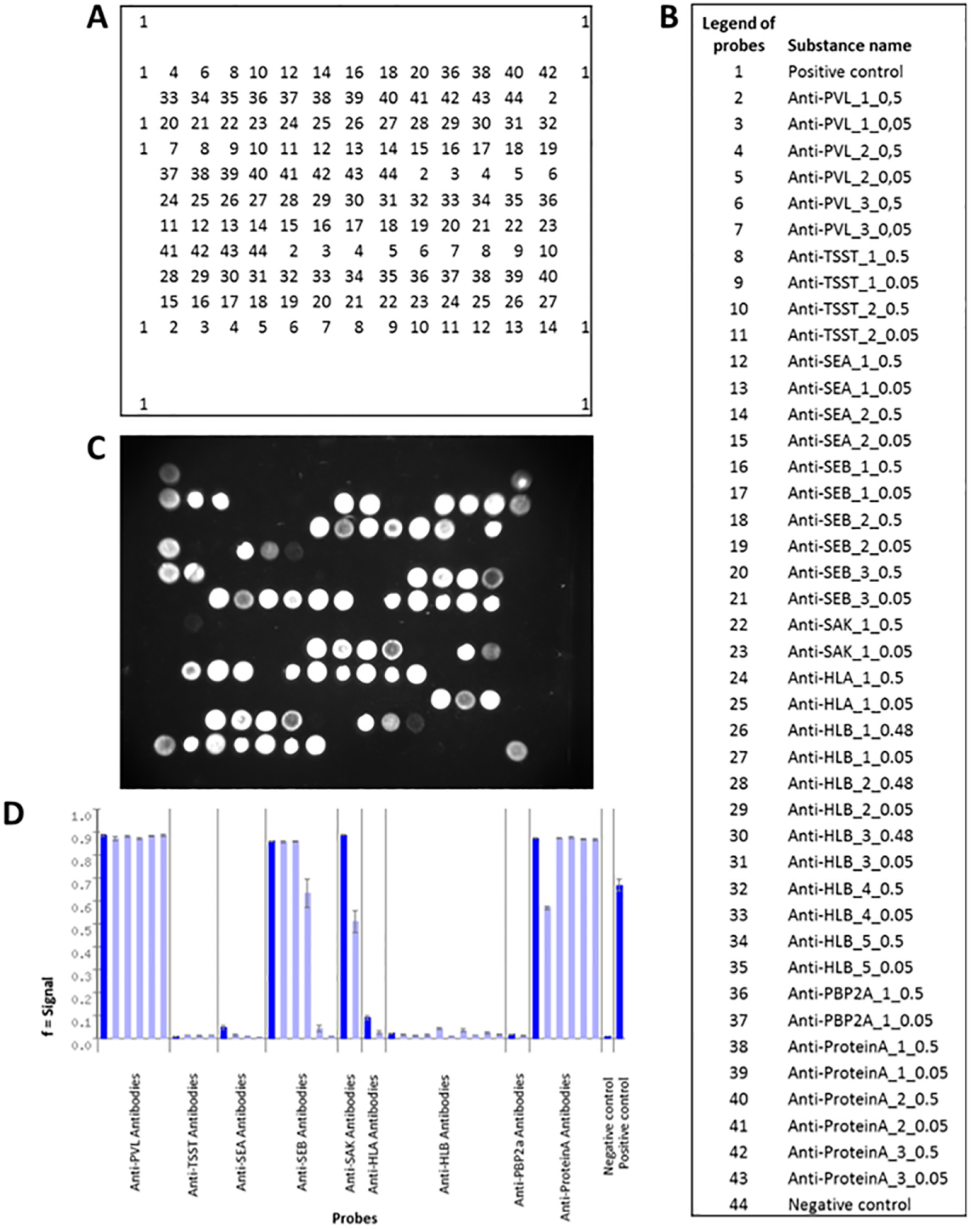
Layout of the protein microarray. (A) Positions of each substance on the chip. (B) Legend to the probes. (C) Picture of a processed fluorescent microarray. (D) Bar graph with signal intensities for the expressed proteins.

Monoclonal antibodies (AB) for the targets PBP2a, SAK, HLB, HLA, SEB, SEA, TSST and lukF-PV were generated via phage display [47] as previously described [44],[21],[25]. SPA antibodies originated from three polyclonal chicken sera (courtesy of Alere Scarborough/Binax). First, all antibodies were screened to find the optimal (*i*.*e*., most specific and sensitive) combination of capture and detection antibodies for each target [44],[21],[25]. Then, all selected antibodies were tested in a mixture to find optimal conditions with regard to both, usage and functionality. The capture antibodies were spotted onto the microarrays, each 3–4 times redundantly and in two different concentrations, 0.5 mg/ml and 0.05 mg/ml, respectively. The use of different concentrations aimed at minimizing effects of steric interference. In order to reach a total protein concentration of 0.5 mg/ml (which is required for the spotting procedure), bovine serum albumin was added (0.45 mg/ml) to the antibodies at higher dilutions. For the number of spotted antibodies per target see Fig 1. For each target, a secondary detection antibody was labelled using Sulf-NHS-LC-Biotin (Pierce, Bonn, Germany) according to the manufacturer’s instructions. A mixture of all 9 biotin-labelled detection antibodies was prepared. The final concentrations of the antibodies were as follows: anti-lukF-PV, 0.2 ng/μl; anti-PBP2a, 0.02 ng/μl; anti-Protein A, 0.5 ng/μl; anti-TSST, 0.1 ng/μl; anti-SEA, 0.05 ng/μl; anti-SEB, 0.05ng/μl; anti-SAK, 0.1 ng/μl; anti-HLA, 0.2 ng/μl; and anti-HLB, 0.07 ng/μl.

### Array procedures

500 μl washing buffer (Protein Binding Kit, Alere Technologies GmbH, Jena, Germany) was added to the arrays and incubated at 37°C and 400 rpm on a shaker for 5 min. 100 μl of blocking solution (Protein Binding Kit, Alere Technologies GmbH) was then added and incubated at 37°C and 300 rpm for 5 min. Meanwhile, culture suspensions in PBS and NaOH (as described above) were prepared. Both culture suspensions were diluted 1:10 in the mixture of biotinylated detection antibodies, and subsequently incubated at 37°C and 400 rpm for 10 min in a separate reaction tube. First, 50 μl of the NaOH-treated sample was added to the array and incubated at 37°C and 300 rpm for 5 min, then the sample was removed and 50 μl of the sample aliquot that was treated with PBS was added and incubated at 37°C and 300 rpm for additional 25 min. The array was then washed with 500 μl washing buffer (5 min, 37°C, 400 rpm). For the detection of specifically bound proteins, 10 μl of purpose-made fluorescent beads (Alere Technologies GmbH), labelled with Cy3 and streptavidin, were added to the array and incubated at 37°C and 300 rpm for 30 min. The arrays were then washed again with 500 μl washing buffer (5 min, 37°C, 400 rpm). The washing buffer was replaced by 100 μl fresh washing buffer and images were taken by a purpose-made reading device, the ATR-Fluo-Reader (Alere Technologies GmbH; http://alere-technologies.com/en/products/lab-solutions/reader-systems/atr-fluo-reader.html; Ehricht et al., 2014).

### Analysis

The images taken by the ATR-Fluo-Reader at exposure times of 500 ms and 1,000 ms, were transferred to a computer for further analysis. Iconoclust software (Alere Technologies GmbH) was used according to manufacturer’s instructions with an assay-specific script. The signals (*S*) of the spots were determined by: *S = M-BG* whereby *M* is the average intensity of the spot and *BG* the intensity of the local background. Thus, the signals range between 0 (negative) and 1 (maximum signal). For each target, specific cut-off values were determined (Table 1). Resulting signal values were considered positive if previously determined cut-offs were reached or surpassed. A software tool was generated for the automatic analysis of the data resulting from the fluorescent microarrays. This software provides reports giving information on the presence of tested proteins based on the previously determined cut-offs. Additionally, results were manually compared to results of the DNA array based genotyping of the respective isolates.

**Table 1:** Specific cut-off values for each target.

Target	Cut off
Positive control	> 0.25
Negative control	< 0.1
lukF-P83	Anti-PVL_2 > 0.2 *
lukF-PV	at least 2 anti-PVL antibodies > 0.2
Protein A	at least 1 anti-Protein A antibody > 0.1
PBP2a	at least 1 anti-PBP2a antibody > 0.2
TSST	at least 1 anti-TSST antibody > 0.2
SEA	at least 1 anti SEA antibody > 0.2
SEB	at least 1 anti-SEB antibody > 0.2
HLA	at least 1 anti-HLA antibody > 0.2
HLB	at least 1 anti-HLB antibody > 0.2
SAK	At least 1 anti-SAK antibody > 0.2

*Anti-PVL_2 Antibody detects exclusive epitopes of lukF-P83. Positive signals for two of the anti-PVL antibodies, 1, 2 or 3, indicate for the target lukF-PV.

## Results

### Assay optimization

The protocol was optimized to facilitate rapid detection of all nine targets simultaneously. Starting from clonal cultures, the bacterial material was inoculated into different solutions to find optimal conditions for the detection of all different membrane and secreted proteins within one and the same assay. NaOH and PBS treated cells were initially tested separately and afterwards in combination. This resulted in partially different performances for the single proteins. After the NaOH treatment, the targets PBP2a, HLA, HLB, SEA, SEB, SAK, protein A and TSST were detected. However, lukF-PV as well as lukF-P83 were not reliably detectable when treating known lukF-PV or lukF-P83 positive strains/isolates with NaOH. In the PBS buffer suspension, all requested targets except PBP2a were detectable.

The protocol was optimized as follows to detect all expressed targets simultaneously: Bacterial culture material was harvested from Columbia blood agar plates (incubated for 18- 24h, 37°C) and divided into two aliquots: one in 1x PBS and one in 100 mM NaOH, respectively. Subsequently, a buffer (see Material/Methods) was added to the NaOH suspension for neutralization. Then, both suspensions were added one after the other to the same array for specific binding of all antigens to their corresponding antibodies (see Methods).

Dilution series with known concentrations of each target (data not shown but can be provided upon request) resulted in following limits of detection: PVL: 0.5 ng/ml, TSST: 0.05 ng/ml, HLA: 0.1 ng/ml, HLB: 0.1 ng/ml, SEA: 0.01 ng/ml, SEB: 0.01 ng/ml, SAK: 0.05 ng/ml and PBP2a: 0.1 ng/ml. No data were obtained for SPA because SPA detection antibodies were polyclonal. In addition, different staphylococcal strains might present with different detection limits because of the presence of multiple binding sites per SPA molecule that might vary in both number and affinity. The use of culture material directly from agar plates ensured that the antigen concentrations was much higher than the detection limit.

### Screening of staphylococcal strains using the protein array and the DNA genotyping array

The results of the antibody arrays and a comparison to the genotypic characterization are summarized in Table 2. 110 isolates and strains were tested using microarrays, the antibody array for phenotyping and the DNA array for genotyping, in parallel. The results of both arrays showed a general concordance of phenotype and genotype for all 110 tested isolates/strains (Table 2). The tested isolates represented diverse clonal complexes (CC1, CC5, CC8, CC9, CC25, CC30, CC 121, CC130, CC133, CC152, CC361, CC479, CC705) from different origins and included MSSA and MRSA.

**Table 2:** Comparison of genotyping data and fluorescent protein microarray results for various staphylococcal isolates.

№	ID	typing	Protocol	SPA		mecA/ PBP2a		PVL		TST		SEA		SEB		SAK		HLA		HLB	
				D	Pr	D	Pr	D	Pr	D	Pr	D	Pr	D	Pr	D	Pr	D	Pr	D	Pr
1	160886	CC1-MSSA	NaOH/PBS	P	P	N	N	P	P	P	P	P^1^	P	N	N	P	P	P	P	T	N
2	199573	CC1-MSSA	NaOH/PBS	P	P	N	N	N	N	N	N	P	P	N	N	P	P	P	P	T	N
3	215392	CC1-MSSA	NaOH/PBS	P	P	N	N	N	N	N	N	P^1^	N	N	N	P	P	P	N	T	N
4	173633	CC1-MSSA	NaOH	P	P	N	N	N	N	N	N	P^1^	P	N	N	P	P	P	P	T	N
5	Sanger476	CC1-MSSA-SCCfus	NaOH	P	P	N	N	N	N	N	N	P^1^	P	N	N	P	P	P	N	P	N
6	231545	CC1-MSSA-SCCfus	NaOH/PBS	P	P	N	N	N	N	N	N	P	P	P	P	P	P	P	P	T	N
7	231549	CC1-MSSA-SCCfus	NaOH/PBS	P	P	N	N	N	N	N	N	P	P	P	P	P	P	P	P	T	N
8	231554	CC1-MSSA-SCCfus	NaOH/PBS	P	P	N	N	N	N	N	N	P	P	P	P	P	P	P	P	T	N
9	MW2	CC1-MRSA-IV [PVL+], USA400	NaOH/PBS	P	P	P	P	P	P	N	N	P	P	N	N	P	P	P	N	T	N
10	124931	CC1-MRSA-IV+SCCfus [PVL+]	NaOH/PBS	P	P	P	P	P	P	N	N	P^1^	P	N	N	P	P	P	P	T	N
11	N315	CC5-MRSA-II, New York-Japan Clone	NaOH	P	P	P	P	N	N	P	P	P^2^	N	N	N	P	P	P	P	T	N
12	ATCC700699	CC5-MRSA-II, New York-Japan Clone	NaOH/PBS	P	P	P	P	N	N	P	P	P^1^	P	N	N	P	P	P	N	T	N
13	223878	CC5-MSSA with a truncated SCC element	NaOH	P	P	N	N	N	N	N	N	P^1^	P	N	N	P	P	P	P	T	N
14	241318	CC5-MRSA-IV, Paediatric clone [PVL+]	NaOH/PBS	P	P	P	P	P	P	N	N	P^2^	N	N	N	P	P	P	A	T	N
15	254809	CC5-MRSA-V+SCCfus	NaOH/PBS	P	P	P	P	N	N	N	N	N	N	P	P	P	P	P	A	A	N
16	NCTC8325	CC8-MSSA	NaOH	P	P	N	N	N	N	N	N	N	N	N	N	P	P	P	P	T	N
17	199572	CC8-MSSA	NaOH/PBS	P	P	N	N	N	N	N	N	P	P	N	N	P	P	P	N	T	N
18	FPR3757	CC8-MRSA-IV (PVL+/ACME+), USA300	NaOH/PBS	P	P	P	P	P	P	N	N	N	N	N	N	P	P	P	P	T	N
19	200914	CC8-MRSA-IV (PVL+/ACME+), USA300	NaOH/PBS	P	P	P	P	P	P	N	N	N	N	N	N	P	P	P	P	T	N
20	200915	CC8-MRSA-IV (PVL+/ACME+), USA300	NaOH/PBS	P	P	P	P	P	P	N	N	N	N	N	N	P	P	P	P	T	N
21	200917	CC8-MRSA-IV (PVL+/ACME+), USA300	NaOH/PBS	P	P	P	P	P	P	N	N	N	N	N	N	P	P	P	P	T	N
22	205975	CC8-MRSA-IV [PVL+/ACME+], USA300	NaOH/PBS	P	P	P	P	P	P	N	N	N	N	N	N	P	P	P	P	T	N
23	227497	CC8-MRSA-IV (PVL+/ACME+), USA300	NaOH	P	P	P	P	P	P	N	N	N	N	N	N	P	P	P	P	T	N
24	176042	CC8-MRSA-IV [PVL+/ACME+], USA300	NaOH/PBS	P	P	P	P	P	P	N	N	N	N	N	N	P	P	P	P	T	N
25	200916	CC8-MRSA-IV (PVL+/ACME+), USA300	NaOH/PBS	P	P	P	P	P	P	N	N	N	N	N	N	P	P	P	N	T	N
26	200918	CC8-MRSA-IV (PVL+/ACME+), USA300	NaOH/PBS	P	P	P	P	P	P	N	N	N	N	N	N	P	P	P	N	T	N
27	200919	CC8-MRSA-IV (PVL+/ACME+), USA300	NaOH/PBS	P	P	P	P	P	P	N	N	N	N	N	N	P	P	P	N	T	N
29	124407	CC8-MRSA-IV (PVL+/ACME+), USA300	NaOH	P	P	P	P	P	P	N	N	N	N	N	N	P	P	P	P	T	N
30	COL	CC8/ST250-MRSA-I, Early/Ancestral MRSA	NaOH/PBS	P	P	P	P	N	N	N	N	N	N	P	P	N	N	P	A	P	N
31	150242	CC9-MRSA-IX	NaOH	P	P	P	P	N	N	N	N	N	N	N	N	N	N	P	N	P	N
32	199027	CC25-MSSA	NaOH/PBS	P	P	N	N	N	N	N	N	N	N	N	N	P	P	P	N	T	N
33	199571	CC30-MSSA	NaOH	P	P	N	N	N	N	P	P	P	P	N	N	P	P	P	N	T	N
34	254033	CC30-MSSA	NaOH/PBS	P	P	N	N	N	N	P	P	P	P	N	N	P	P	P	N	T	N
35	199575	CC30-MSSA	NaOH	P	P	N	N	N	N	P	P	P	P	N	N	P	P	P	N	T	N
36	199576	CC30-MSSA	NaOH	P	P	N	N	N	N	P	P	P^2^	N	N	N	P	P	P	N	T	N
37	252785	CC30-MSSA	NaOH/PBS	P	P	N	N	N	N	P	P	P	P	N	N	P	P	P	N	T	N
38	254801	CC30-MRSA	NaOH/PBS	P	P	P	P	P	P	N	N	P	P	N	N	P	P	P	N	P	N
39	198848	CC30-MRSA-IV [PVL+]	NaOH/PBS	P	P	P	P	P	P	N	N	N	N	N	N	P	P	P	N	P	N
40	234946	CC30-MSSA, [lukF-P83/lukM+]	NaOH/PBS	P	P	N	N	P^b^	N	N	N	N	N	N	N	N	N	P	N	P	N
41	200922	CC30-MRSA-IV-SCCfus [PVL+]	NaOH/PBS	P	P	P	P	P	P	P	P	N	N	N	N	P	P	P	N	T	N
42	200923	CC30-MRSA-IV-SCCfus [PVL+]	NaOH/PBS	P	P	P	P	P	P	P	P	N	N	N	N	N	N	P	N	P	N
43	200924	CC30-MRSA-IV-SCCfus [PVL+]	NaOH/PBS	P	P	P	P	P	P	P	P	N	N	N	N	P	P	P	N	P	N
44	215384	CC30-MRSA-IV	NaOH	P	P	P	P	N	N	P	P	N	N	N	N	P	N	P	N	T	N
45	200920	CC30-MRSA-IV [PVL+], Southwest Pacific Clone	NaOH/PBS	P	P	P	P	P	P	N	N	N	N	N	N	P	P	P	N	T	N
46	200921	CC30-MRSA-IV [PVL+], Southwest Pacific Clone	NaOH/PBS	P	P	P	P	P	P	N	N	P	P	N	N	P	P	P	N	T	N
47	200925	CC30-MRSA-IV [PVL+], Southwest Pacific Clone	NaOH/PBS	P	P	P	P	P	P	N	N	P	P	N	N	P	P	P	N	P	N
48	Sanger252	ST36/39-MRSA-II, UK-EMRSA-16	NaOH/PBS	P	P	P	P	N	N	N	N	P^1^	P	N	N	P	P	P	N	T	N
49	236783	CC88-MRSA-IV [PVL+], [lukF-PV+, lukS-PV-]	NaOH/PBS	P	P	P	P	P^4^	N	N	N	P^2^	N	N	N	P	P	P	N	T	N
50	238233	CC121-MSSA [PVL+]	NaOH/PBS	P	P	N	N	P	P	N	N	N	N	N	N	P	P	P	N	T	N
51	238234	CC121-MSSA [PVL+]	NaOH/PBS	P	P	N	N	P	P	N	N	N	N	N	N	P	P	P	N	T	N
52	238235	CC121-MSSA [PVL+]	NaOH/PBS	P	P	N	N	P	P	N	N	N	N	N	N	P	P	P	N	T	N
53	238236	CC121-MSSA [PVL+]	NaOH/PBS	P	P	N	N	P	P	N	N	N	N	P	P	P	P	P	N	T	N
54	238237	CC121-MSSA [PVL+]	NaOH/PBS	P	P	N	N	P	P	N	N	N	N	P	A	P	P	P	N	T	N
55	238238	CC121-MSSA [PVL+]	NaOH/PBS	P	P	N	N	P	P	N	N	N	N	P	P	P	P	P	N	T	N
56	238239	CC121-MSSA [PVL+]	NaOH/PBS	P	P	N	N	P	P	N	N	N	N	P	P	P	P	P	N	T	N
57	238240	CC121-MSSA [PVL+]	NaOH/PBS	P	P	N	N	P	P	N	N	N	N	P	P	P	P	P	N	T	N
58	238241	CC121-MSSA [PVL+]	NaOH/PBS	P	P	N	N	P	P	N	N	N	N	P	P	P	P	P	N	T	N
59	238242	CC121-MSSA [PVL+]	NaOH/PBS	P	P	N	N	P	P	N	N	N	N	P	A	P	P	P	N	T	N
60	238243	CC121-MSSA [PVL+]	NaOH/PBS	P	P	N	N	P	P	N	N	N	N	P	A	P	P	P	N	T	N
61	238244	CC121-MSSA [PVL+]	NaOH/PBS	P	P	N	N	P	P	N	N	N	N	P	P	P	P	P	N	T	N
62	238245	CC121-MSSA [PVL+]	NaOH/PBS	P	P	N	N	P	P	N	N	N	N	P	P	P	P	P	N	T	N
63	238246	CC121-MSSA [PVL+]	NaOH/PBS	P	P	N	N	P	P	N	N	P^2^	N	P	P	P	P	P	N	T	N
64	238247	CC121-MSSA [PVL+]	NaOH/PBS	P	P	N	N	P	P	N	N	N	N	P	P	P	P	P	N	T	N
65	238248	CC121-MSSA [PVL+]	NaOH/PBS	P	P	N	N	P	P	N	N	N	N	P	P	P	P	P	N	T	N
66	238249	CC121-MSSA [PVL+]	NaOH/PBS	P	P	N	N	P	P	N	N	N	N	N	N	P	P	P	N	T	N
67	238251	CC121-MSSA [PVL+]	NaOH/PBS	P	P	N	N	P	P	N	N	N	N	P	A	P	P	P	N	T	N
68	238252	CC121-MSSA [PVL+]	NaOH/PBS	P	P	N	N	P	P	N	N	N	N	P	A	P	P	P	N	T	N
69	238253	CC121-MSSA [PVL+]	NaOH/PBS	P	P	N	N	P	P	N	N	N	N	P	A	P	P	P	N	T	N
70	253431	CC121-MSSA [PVL+]	NaOH/PBS	P	P	N	N	P	P	N	N	N	N	P	P	P	P	P	N	T	N
71	238254	CC121-MSSA	NaOH/PBS	P	P	N	N	N	N	N	N	N	N	N	N	P	P	P	N	T	N
72	238255	CC121-MSSA	NaOH/PBS	P	P	N	N	N	N	N	N	N	N	N	N	P	P	P	N	T	N
73	238256	CC121-MSSA	NaOH/PBS	P	P	N	N	N	N	N	N	P^2^	N	N	N	P	P	P	N	T	N
74	224815	CC130-MRSA-XI [mecC +]	NaOH	P	P	N	N	N	N	N	N	N	N	N	N	N	N	P	N	P	P
75	225779	CC130-MRSA-XI, [mecC +]	NaOH	P	P	N	N	N	N	N	N	N	N	N	N	N	N	P	N	P	P
76	124627	CC133-MSSA [lukF-P83/lukM+]	NaOH/PBS	P	P	N	N	P^b^	P^b^	P	P	P^3^	P	N	N	N	N	P	N	P	P
77	215375	CC133-MSSA, [lukF-P83/lukM+]	NaOH/PBS	P	P	N	N	P^b^	P^b^	P	P	N	N	N	N	N	N	P	N	P	P
78	252784	CC133-MSSA [lukF-P83/lukM+]	NaOH/PBS	P	P	N	N	P^b^	P^b^	P	P	N	N	P^X^	N	N	N	P	N	P	P
79	215373	CC133-MSSA, [lukF-P83/lukM+]	NaOH/PBS	P	P	N	N	P^b^	N	N	N	P^3^	P	N	N	P	P	P	N	P	N
80	238250	CC152-MSSA [PVL+]	NaOH/PBS	P	P	N	N	P	P	N	N	N	N	N	N	P	P	P	N	P	P
81	124474	CC361-MRSA-IV, WA MRSA-29	NaOH/PBS	P	P	P	P	N	N	P	P	N	N	P	P	P	P	P	N	T	N
82	225776	CC398-MRSA-IV	NaOH	P	P	P	P	N	N	N	N	N	N	N	N	N	N	P	N	P	P
83	199873	CC398-MRSA-V	NaOH	P	P	P	P	N	N	N	N	N	N	N	N	N	N	P	N	P	N
84	199874	CC398-MRSA-V	NaOH	P	P	P	P	N	N	N	N	N	N	N	N	N	N	P	N	P	N
85	225778	CC398-MRSA-V	NaOH	P	P	P	P	N	N	N	N	N	N	N	N	N	N	P	N	P	P
86	210067	CC479-MSSA [lukF-P83/lukM+]	NaOH/PBS	P	A	N	N	P^b^	P^b^	N	N	N	N	N	N	N	N	P	N	P	P
87	178400	CC479-MSSA [lukF-P83/lukM+]	NaOH/PBS	P	N	N	N	P^b^	P^b^	N	N	N	N	N	N	N	N	P	N	P	P
88	138830	CC479-MSSA [lukF-P83/lukM+]	NaOH/PBS	P	P	N	N	P^b^	A^b^	N	N	N	N	N	N	N	N	P	N	P	P
89	138831	CC479-MSSA [lukF-P83/lukM+]	NaOH/PBS	P	P	N	N	P^b^	A^b^	N	N	N	N	N	N	N	N	P	N	P	P
90	RF122	CC705-MSSA [lukF-P83/lukM+]	NaOH/PBS	P	P	N	N	P^b^	A	P	P	N	N	N	N	N	N	P	N	P	P
91	196911	CC705-MSSA [lukF-P83/lukM+]	NaOH/PBS	P	P	N	N	P^b^	P^b^	P	P	N	N	N	N	N	N	P	N	P	P
92	196916	CC705-MSSA [lukF-P83/lukM+]	NaOH/PBS	P	P	N	N	P^b^	P^b^	N	N	N	N	N	N	N	N	P	N	P	P
93	119087	CC705-MSSA [lukF-P83/lukM+]	NaOH	P	P	N	N	P^b^	P^b^	N	N	N	N	N	N	N	N	P	P	P	P
94	138826	CC705-MSSA [lukF-P83/lukM+]	NaOH/PBS	P	A	N	N	P^b^	P^b^	P	P	N	N	N	N	N	N	P	N	P	P
95	101818	ST8-MRSA-IIB, Irish AR13/14	NaOH/PBS	P	P	P	P	N	N	N	N	P^1^	P	N	N	P	P	P	N	T	N
96	124414	ST59-MRSA-IV+V, WA MRSA-15	NaOH/PBS	P	P	P	P	N	N	N	N	P^1^	P	P	P	P	P	P	N	P	P
97	215382	ST80-MRSA-IV [PVL+], “European Clone”	NaOH	P	P	P	P	P	P	N	N	N	N	N	N	P	P	P	N	T	N
98	224519	ST239-MRSA-III agr/hld deletion variant	NaOH/PBS	P	P	P	P	N	N	N	N	P	P	N	N	P	P	P	N	T	N
99	200926	ST772-MRSA-V [PVL+], Bengal Bay Clone/WA MRSA-60	NaOH/PBS	P	P	P	P	P	P	N	N	P	P	N	N	N	N	P	N	T	N
100	238824	ST772-MRSA-V [PVL+], Bengal Bay Clone/WA MRSA-60	NaOH/PBS	P	P	P	P	P	P	N	N	P	P	N	A	N	N	P	N	T	N
101	200930	ST772-MRSA-V [PVL+], Bengal Bay Clone/WA MRSA-60	NaOH/PBS	P	P	P	P	P	P	N	N	P	P	N	N	N	N	P	N	A	N
102	145330	ST772-MRSA-V [PVL+], Bengal Bay Clone/WA MRSA-60	NaOH/PBS	P	P	P	P	P	P	N	N	P^1^	P	N	N	N	N	P	N	T	N
103	200927	ST772-MRSA-V [PVL+], Bengal Bay Clone/WA MRSA-60	NaOH/PBS	P	P	P	P	P	P	N	N	P	P	N	N	N	N	P	N	T	N
104	200928	ST772-MRSA-V [PVL+], Bengal Bay Clone/WA MRSA-60	NaOH/PBS	P	P	P	P	P	P	N	N	P	P	N	N	N	N	P	N	A	N
105	200929	ST772-MRSA-V [PVL+], Bengal Bay Clone/WA MRSA-60	NaOH/PBS	P	P	P	P	P	P	N	N	P	P	N	N	N	N	P	N	A	N
106	200931	ST772-MRSA-V	NaOH/PBS	P	P	P	P	N	N	N	N	N	N	N	N	N	N	P	N	A	N
107	ATCC35984	*S*. *epidermidis*, [mecA+]	NaOH	N	N	P	P	N	N	N	N	N	N	N	N	N	N	N	N	N	N
108	ATCC12228	*S*. *epidermidis*	NaOH	N	N	N	N	N	N	N	N	N	N	N	N	N	N	N	N	N	N
109	225781	*S*. *sciuri*	NaOH	N	N	N	N	N	N	N	N	N	N	N	N	N	N	N	N	N	N
110	223885	*S*. *capitis*	NaOH	N	N	N	N	N	N	N	N	N	N	N	N	P^X^	N	N	N	N	N
111	BL21-DE3	*E*. *coli*	NaOH	N	N	N	N	N	N	N	N	N	N	N	N	N	N	N	N	N	N

^1^ = *sea*-FRI100;

^2^ = *sea*-N315;

^3^ = *sea*320;

^4^ = *lukF*-PV+/*lukS*-PV-;

^A^ = ambiguous;

^B^ = *lukF*-P83/*lukM*;

^D^ = DNA;

^N^ = negative;

^P^ = positive;

^Pr^ = Protein;

^T^ = truncated;

^X^ = one DNA probe out of 2 only

In addition to the *S*. *aureus* isolates, an *E*. *coli* strain (BL21-DE3) and one *S*. *capitis* isolate were tested on both arrays as negative controls. For these strains, none of the target genes and none of the corresponding proteins were detectable. Two *S*. *epidermidis* strains (ATCC35984 and ATCC12228) were also tested. The strain ATCC35984 harbored the *mecA* gene. This gene, as well as the corresponding protein PBP2a, was correctly identified. Strain ATCC12228 was negative for all tested targets.

### Identification and detection of variants of targets

For three targets, distinct variants are known. These included *mecA*/PBP2a, PVL and SEA.

In addition to *mecA*, there is “*mecA1*” in various animal staphylococci that does not confer resistance to methicillin, as well as *mecC*. In a “*mecA1*”-positive/*mecA*-negative *S*. *sciuri* isolate, no PBP2a was detected. Two isolates of CC130-MRSA-XI that carried the *mecC* gene as part of a SCC*mec* XI element did not yield signals with PBP2a antibodies. That indicates a specific recognition of *mecA* encoded PBP2a by the antibody and no cross-reaction with the gene products of “*mecA1”* or *mecC*.

In *lukF-P83/lukM*-positive *S*. *aureus* isolates, lukF-P83 was detected by one of the three PVL antibodies, thus facilitating both its detection, as well as its differentiation from PVL.

For SEA, different alleles can be recognized that will be designated here *sea*-FRI100 (GenBank accession number L22565.1 [482…526]), *sea*-320 (GenBank accession number CP001996.1 [1144119…1144898]) and *sea*-N315 (GenBank accession number BA000018.3 [2011380…2012153]). Several *S*. *aureus* isolates with all of these *sea* alleles were tested. Anti-SEA-antibodies detected SEA corresponding to alleles *sea*-FRI100 and *sea*-320, whereas SEA of allele *sea*-N315 was not identified.

## Discussion

The multiplex protein microarray as described herein is a test for the fast and direct detection of relevant staphylococcal proteins from clonal culture material. These include PBP2a, allowing the use of the assay as a confirmatory test for the identification of MRSA after obtaining a doubtful susceptibility test result. In addition, the *S*. *aureus* species marker SPA [48], [49] and several clinically relevant toxins are included, *i*.*e*., PVL, TSST, HLA, HLB and enterotoxins A and B. No simple non-molecular assays such as ELISAs, regardless whether using single- or multiplex assays, are currently available in routine settings.

Currently, there is a set of genotypic methods in bacterial routine diagnostics for strain identification and determination of relevant virulence and resistance markers. Common techniques are PCR or DNA microarray hybridization. Most of these assays give information about the presence of species-specific virulence or resistance genes, but not about their expression. For protein expression and functionality, molecular phenotypic techniques are well established. ELISAs, lateral flow tests for protein detection in general, or automated micro dilution techniques, with regard to resistance markers, are current methods. A main disadvantage of ELISAs or LFs is that only one, or a low number of targets can be tested in one experiment. The multiplex protein array developed herein allows a rapid, parallel and economic performance to detect several markers within a single experiment. A pure culture harvested from a blood agar plate can be used to perform the assay. These are optimal preconditions for integrating the assay into routine laboratories.

The array contains antibodies against epitopes of protein A. This cell wall located protein is used in this assay as species marker for *S*. *aureus*. All *S*. *aureus* isolates tested in this study gave positive signals for SPA. By contrast, the tested coagulase-negative staphylococci (CoNS), *S*. *epidermidis*, *S*. *sciuri and S*. *capitis*, as well as another control strain, *E*. *coli*, tested negative for this *S*. *aureus* species-specific marker. Therefore, these anti-protein A-antibodies act as positive controls for *S*. *aureus* isolates in the experiments.

The qualitative detection of proteins using the antibody microarray was verified by checking the presence of corresponding genes using the DNA microarray. Generally, the results described herein showed very good concordance of genotypic and phenotypic results.

The protein microarray allows the detection of PBP2a, which is related to methicillin resistance in tested isolates. Thus, the protein microarray can be used as a confirmatory assay after methicillin resistance detection in microdilution or agar diffusion tests. Fast discrimination between MSSA and MRSA plays an important role in hospitals. New patients can be screened and, if necessary (*i*.*e*., in case of a MRSA-positive result), be isolated from other patients to avoid transmissions or even outbreaks. In addition, appropriate treatment can be initiated. Recently, a new *mecA* homologue, *mecC*, was discovered. It is located on a novel SCC*mec* element type XI and also confers resistance to methicillin [7],[8]. This *mecC* encoded protein could not be detected using the assay described herein. The antibodies currently used are specific for *mecA*-encoded PBP2a and do not cross-react with the gene product of *mecC*. We did not aim to cover *mecC* at this stage. The reason being a very low prevalence that can be estimated to be in an order of magnitude of about 0.1% to 1% of MRSA isolates only [9],[50]. However, in case of an increasing prevalence, it would be feasible to add an antibody that recognizes the gene product of *mecC*. *S*. *sciuri* harbors “*mecA1*”, *i*.*e*., one of several deviant *mecA* alleles that do not confer beta lactam resistance [10–11]. One *S*. *sciuri* isolate was tested which did not yield a signal for PBP2a. This indicates that possible *S*. *sciuri* contaminants would not interfere with the *mecA*/PBP2a detection.

PVL is a marker for virulent *S*. *aureus* strains including CA-MRSA. A guideline was recently issued in the UK for the management of patients infected with PVL-positive *S*. *aureus* (https://www.gov.uk/government/uploads/system/uploads/attachment_data/file/322363/PVL_LRTI_risk_assessment_protocol.pdf). For the implementation of this guideline, a diagnostic test for the detection of PVL is necessary. Currently, testing for the presence of PVL is performed in molecular/reference laboratories. The assay described herein provides the opportunity to rapidly detect PVL in routine settings without the use of PCR techniques, and therefore to adequately and timely manage patients with PVL-positive *S*. *aureus*. An increasing prevalence of strains that harbor both PVL and PBP2a, was observed [51], and this assay is the first available, non-molecular test able to detect both relevant proteins in parallel.

In veterinary medicine, leukocidins are also relevant virulence factors. The leukocidin lukF-P83/lukM is associated with veterinary disease such as bovine mastitis. It appears to be a marker for epidemic strains within a herd [23]. The protein microarray might be a useful phenotypic test in veterinary medicine to differentiate between lukF-P83/lukM-positive and—negative strains. Its detection could help to distinguish an isolated, sporadic case, following a transmission from the farmer, or secondary to an injury from an infection caused by a virulent strain with potential for epidemic spread within a herd [23]. Isolates of CC479 and CC705, harboring the genes *lukF-P83/lukM*, also showed positive signals for lukF-P83 expression.

Rapid detection of staphylococcal enterotoxins A and B is advisable in the case of foodborne intoxication. Their detection is helpful for epidemiological monitoring of such intoxications and might help to elucidate outbreaks. Currently, cultured isolates can be screened for the production of enterotoxins A and B. Antibodies to additional enterotoxins could be generated and added at a later stage, and protocols for the direct detection of enterotoxins in suspected food should be developed and evaluated.

Rapid detection of TSST in clinical isolates might also be helpful. For instance, cases of toxic shock syndrome can superficially resemble Lyell syndrome. Toxic shock syndrome requires aggressive antibiotic chemotherapy, whereas in Lyell syndrome discontinuation of any unnecessary medication is warranted. A rapid assay for detection of TSST production from *S*. *aureus* primary cultures could facilitate a rapid decision, thereby aiding management of patients with these severe conditions.

The *hla* gene was present in all isolates tested and therefore can be regarded as a potential species-specific marker for *S*. *aureus*. However, for alpha toxin, major discrepancies between the assays for the gene and the corresponding protein were detected. Results from a previous study were confirmed, that showed that the levels of HLA expression depended on the CC affiliation. Isolates belonging to clonal complexes such as CC1, CC5 and CC8 showed variable to high levels of alpha toxins. Isolates that belonged, *e*.*g*., to CC22, CC30, CC45, CC30, CC479 and CC705 did not yield detectable levels of alpha toxin under the given culture conditions despite their proven carriage of the *hla* gene. A reason for this discrepancy is not yet known. It might be related to *in vitro* conditions and/or different gene regulation mechanisms [25].

The *sak*-carrying bacteriophages are usually present in *S*. *aureus* from humans where they are inserted into the *hlb* coding sequence. SAK will be expressed in humans, whereas HLB is mainly expressed in animals. The results of this study confirm these previous findings. The livestock-associated strains of CC398, CC479 or CC705 express HLB but do not carry SAK. Human *S*. *aureus* isolates showed converse expression of both these proteins. The expression of either SAK or HLB can be distinguished by the multiplex array, and is more of a scientific than a diagnostic interest.

The current assay provides for a wide range of future developments and applications. The fluorescent array image results showed characteristic expression patterns for several strains. An expanded assay could use these typical “fingerprints” for presumptive strain identification. Several strains are characterized in detail by carrying different resistance and virulence genes. For example, *S*. *aureus* strain USA300 (ST8-MRSA-IV/PVL+/ACME+) carries the genes *mecA*, *spa* and *sak*, and expresses the corresponding proteins, as well as the exotoxins PVL and alpha toxin. In comparison, the Bengal Bay Clone/WA MRSA-60 (ST772-MRSA-V/PVL+) yields under the given culture conditions PBP2a, SPA, PVL and enterotoxin A, but no alpha toxin and no staphylokinase. Using these different expression patterns, presumptive strain identification can be done directly based on the fluorescent array images. However, use for fingerprinting purposes has limitations that require further study. Firstly, the number of target proteins should be expanded in order to identify more strains. More enterotoxins as well as exfoliative toxins might also be of clinical interest. Secondly, some target proteins are located on mobile genetic elements. Thus, their carriage in different isolates of one strain may vary. Thirdly, their expression might theoretically vary depending on clinical background, including previous medication or culture conditions. Future studies should therefore aim at expansion of the target panel and standardization issues. Additionally, the technology of a multiplex protein microarray can be useful for other microorganisms.

The assay was developed for qualitative analyses of protein expression. However a quantitative analysis of the fluorescent arrays might also be possible. Fluorescence allows an easier determination of protein quantities compared to precipitation staining which was used in previously studies.

As described, there are quantitative differences between several *S*. *aureus* strains with regard to the HLA and PVL expression [25],[21]. Among other factors, the size of the inoculum requires standardization to facilitate a comparison of results from different experiments. The use of a liquid medium is therefore advisable. Further experiments are necessary to establish a suitable medium and a corresponding protocol. For a standardized method, the quantities of individual target proteins need to be normalized with a conserved and constant species marker as an internal standard, with reference to the cell count. In future, quantitative measurements could be used to study strain-specific virulence traits and a possible influence of different antibiotics on the virulence factor expression.

The newly developed assay as described herein allows the multiplex detection of staphylococcal proteins, and contains the option to test the influence of external factors on protein expression. This might be a useful test in routine diagnostics as well as for research purposes.

## Supporting Information

S1 File Full data set of signal intensities for each experiment as obtained by DNA- and protein microarrays. (XLSX)
